# Effectiveness of a nutrition education and counselling training package on antenatal care: a cluster randomized controlled trial in Addis Ababa

**DOI:** 10.1093/heapol/czaa101

**Published:** 2020-11-09

**Authors:** Afrah Mohammedsanni Omer, Demewoz Haile, Bilal Shikur, Erlyn Rachelle Macarayan, Seifu Hagos

**Affiliations:** c1 Department of Nutrition and Dietetics, School of Public Health, College of Health Sciences, Addis Ababa University, Ethiopia; c2 Harvard Global Health Institute, Cambridge, MA 02138, USA; c3 Department of Health Policy and Management, Harvard TH Chan School of Public Health, Boston, MA, USA

**Keywords:** Antenatal care, nutrition education, capacity building, health facilities, health professionals, health services, maternal health, pregnancy, primary health care, randomized controlled trial

## Abstract

The World Health Organization (WHO) recommends the need for a strong nutrition training package for practitioners, including antenatal care (ANC) providers. Without such a training package, ANC visits remain a missed opportunity to address nutritional problems among pregnant women. This study evaluated the effectiveness of an in-service nutrition education and counselling package on the providers’ counselling skills during ANC visits. A cluster randomized controlled trial was conducted in Addis Ababa, Ethiopia. All health-care providers working in ANC units across 20 health centres participated in this study. Health centres were allocated to intervention and control arms using a matched-pair randomization technique. An in-service nutrition education and counselling package, including training for ANC providers, supportive supervision and provision of modules, pamphlets and job aids, was provided for health centres assigned to the intervention arm. Observation checklists were used to assess the counselling skills of health-care providers. We used mixed-effect linear regression to evaluate the impact of the intervention. Significantly more health-care providers in the intervention arm informed pregnant women about the need to have one additional meal (Difference in proportion [DP] 49.17% vs −0.84%; DID 50.0%), about minimum required dietary diversity (DP 72.5% vs −2.5%; DID 75.0%) and about gestational weight gain (DP 68.33% vs −8.33%; DID 76.6%). Furthermore, providers improved in identifying key difficulties that pregnant women face (DP 28.34% vs −2.5%; DID 30.8%), and in recommending simple achievable actions on nutrition during pregnancy (DP 20.8% vs −10.9%; DID 31.6%). The intervention did not have statistically significant effects on how providers informed women about early initiation of breastfeeding (DP 6.67% vs 9.17%; DID −2.5%). The comprehensive in-service nutrition education and counselling package improved how ANC providers engaged with pregnant women and delivered nutrition messages during ANC consultations. This trial was registered in the Pan African Clinical Trial (PACTR registry, PACTR20170900 2477373; Date issued 21 September 2017).

**KEY MESSAGES**

- Malnutrition among women of the reproductive age group (15–49 years) in Ethiopia is relatively high compared with in its neighbouring countries, with 22% having a body mass index of <18.5 kg/m^2^ (undernourished) and 24% with anaemia prevalence. Only 18% of pregnant women in Addis Ababa took the 90+ iron folic acid supplement tablets recommended by the World Health Organization, while 35.6% of pregnant women did not take any supplements throughout their pregnancy.
- To address maternal, as well as other nutritional, concerns in the country, the government of Ethiopia developed a Blended and Integrated Nutrition Learning Module (BINLM) in 2016. However, up to this date, no evaluation has so far been made on the effectiveness of BINLM in improving the nutrition education and counselling skills of antenatal care (ANC) providers in Ethiopia.
- This study found that an in-service nutrition education and counselling package developed by adapting the BINLM improves ANC providers’ approach and the nutrition messages delivered to pregnant women.
- The study recommends provision of comprehensive in-service nutritional training for providers working in ANC units, including supportive supervision and preparation of ANC nutritional guidelines and job aids to provide adequate nutrition education for pregnant women by trained ANC providers.

## Introduction

Malnutrition among women of the reproductive age group (15–49 years) in Ethiopia is relatively high compared with in its neighbouring countries, with 22% of women having a body mass index of <18.5 kg/m^2^ (undernourished) and 24% with anaemia prevalence (The DHS Program ICF Ethiopia, 2016). About 58% of pregnant women in the country did not take iron folic acid (IFA) supplementation during their last pregnancy, and only 5.1% took the recommended 90+ iron tablets (The DHS Program ICF Ethiopia, 2016). Malnutrition among pregnant women may lead to higher risks of stillbirth, miscarriage, low birth weight, mortality, impaired cognitive development and sub-optimal productivity in later life (Wu *et al.*, 2004; Williamson, 2006; Wilkinson and Tolcher, 2010; Veena *et al.*, 2016).

Nutrition education and counselling of pregnant women is critical for every antenatal care (ANC) visit. In Ethiopia, 62% of pregnant women received at least one ANC visit for their last birth, and 66% of these women said they received nutritional education and counselling, but the quality and extent of counselling they received was unknown (The DHS Program ICF Ethiopia, 2016). Without an adequate nutritional education and counselling programme that can be integrated into existing services such as ANC, malnutrition among pregnant women and their children will remain a pressing challenge in Ethiopia and other low- and middle-income countries.

The World Health Organization (WHO) recommends the need for a strong training package for practitioners, which includes a standardized, evidence-based, sustainable, reproducible, accessible and adaptable guidance on nutrition (World Health Organization, 2016). Without such a training package, ANC visits will remain a missed opportunity to address nutritional problems among pregnant women, which is critical for women and their children.

Over the past decades, the government of Ethiopia has emphasized the importance of nutrition interventions in its development plans, national nutrition programmes and ‘1000 days initiatives’—a group of interventions targeted from the first day of conception to the second birthday of a child (Federal Ministry of Health Ethiopia, 2005, 2010, 2013, 2015, 2016b). To address maternal as well as other nutritional concerns in the country, the government of Ethiopia developed a Blended and Integrated Nutrition Learning Module (BINLM), which is a compact disc-based training material introduced in 2016 (Federal Ministry of Health Ethiopia, 2016a).

To this date, no evaluation has been conducted to assess the effectiveness of BINLM in improving the nutrition education and counselling skills of ANC providers in Ethiopia. By building on existing nutritional training programmes such as the BINLM, we developed a comprehensive in-service nutrition education and counselling training package for ANC providers and evaluated the effectiveness of this programme on the providers’ engagement with pregnant women and nutritional counselling skills during ANC visits in primary care units of Addis Ababa, Ethiopia.

## Methods

### Setting

This study was conducted in Addis Ababa, Ethiopia from August to December 2017. Addis Ababa is the capital city of Ethiopia, consisting of 10 sub-cities and 94 health centres (Federal Ministry of Health Ethiopia, 2017). Health centres are the primary health-care units of Addis Ababa. They provide both preventive and curative services and serve as referral centres to hospitals. Health centres are usually staffed by about four health-care providers per ANC unit. Health officers, nurses and midwives are at the forefront of providing ANC services in the health centres. High-risk pregnancies including pregnancies requiring caesarean delivery are referred to catchment hospitals of the health centres to receive ANC and delivery services (Federal Ministry of Health Ethiopia, 2015). Among the city’s 1 134 510 women aged 15–49 years, 81 915 were expected to be pregnant by the year 2018 (Federal Ministry of Health Ethiopia, 2017). The majority (97%) of pregnant women in the city of Addis Ababa receive ANC from skilled providers (The DHS Program ICF Ethiopia, 2016). However, only 18% of pregnant women in Addis Ababa took 90+ IFA supplement tablets recommended by the World Health Organization during their last pregnancy, while 35.6% of pregnant women did not take any supplements (The DHS Program ICF Ethiopia, 2016). Consequently, anaemia among women aged 15–49 years in the city is at 16% (The DHS Program ICF Ethiopia, 2016).

### Study design

We conducted a cluster randomized controlled trial from August 2017 to December 2017, clustering by health centres grouped by each of the 10 sub-cities in Ethiopia. With a total of 94 health centres in Ethiopia, each of the 10 sub-cities had an average of nine health centres. We then excluded 12 health centres (three non-governmental health centres and nine health centres with <50 average monthly caseload for ANC). For the remaining health centres, we randomized health centres by using a matched-pair randomization technique. We randomly selected one health centre from each sub-city and then a matched pair for each health centre was selected from the same sub-city using the following matching criteria:

1. Health centres with a non-adjacent catchment area from the first randomly selected health centre (which is at least one district in between the health centres) from each sub-city to prevent information contamination; and,
2. Health centres within ± 15% range of an average monthly ANC case load from the first randomly selected health centre in each sub-city in order to keep baseline balance.

Data for all matching variables were obtained from the Addis Ababa Health Bureau (AAHB) database, including city maps on which health centres are located and sub-city health information reports. Following a matched-pair randomization scheme, health centres were randomized, with one health centre per sub-city allocated to intervention and the other health centres to control arms. Non-study personnel conducted the randomization by using balloting method. We invited these 20 health centres to participate prior to randomization without disclosure of allocation status. In this study, we did not calculate sample size since we enrolled all ANC providers from the selected health centres. All study participants were aware of their study allocation status (intervention or control) during baseline data collection.

### Intervention: comprehensive in-service nutrition education and counselling training package aimed at improving counselling skills of ANC providers working in ANC units

We adapted and modified the existing BINLM (Module II: Adolescent, Maternal, Infant & Young Child Nutrition [AMIYCN]) of the Federal Ministry of Health in Ethiopia to include additional nutritional messages and counselling items from other international guidelines and documents as well as the use of the health belief model while counselling pregnant women. The health belief model is one of the behavioural change models adapted to explain and predict health behaviours. The model suggests that an individual’s perceived threat of disease or negative outcome is a key determinant of whether he or she adopts a healthy behaviour. It also suggests that the benefits and barriers of changing health behaviour must be taken into consideration, as those who perceive more benefits than barriers are more likely to take action (Taylor *et al.*, 2006; Moore and Johnson, 2015). We then trained all ANC providers of the health centres assigned to the intervention arm of the study (*n* = 40 ANC providers, 10 health centres). Each ANC provider had a two-day training with the adapted BINLM. The first author (AO), who is a certified BINLM trainer, provided the training supported by all other co-authors along with a Health Information Education and Communication programme officer and a nutrition expert from AAHB.

**Figure 1:**
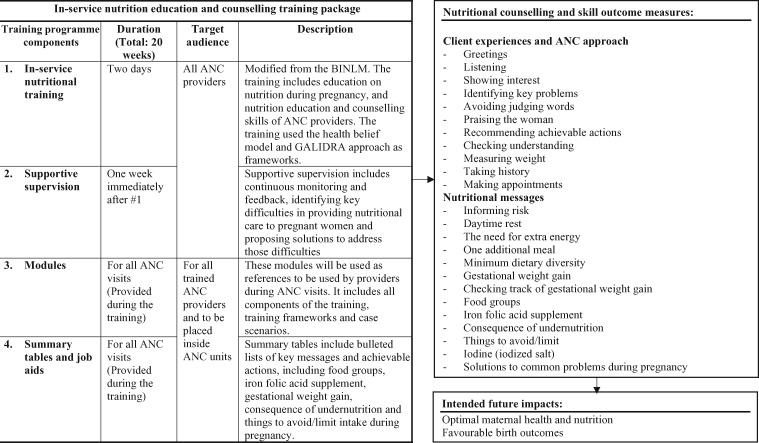
In-service nutrition education and counselling training package. ANC, antenatal care; ANCPs, antenatal care providers; BINLM, Blended and Integrated Nutrition Learning Module; GALIDRA, Greet, Ask, Listen, Identify, Discuss, Repeat, Appoint

The training was composed of three sessions (Figure 1). First, ANC providers reflected on why they needed a nutritional training programme specifically intended for pregnant women. Second, providers discussed nutrition for pregnant women, highlighting women’s nutritional needs during pregnancy, the importance of prescribing IFA supplementation to pregnant women, the importance of adherence to IFA supplements, pregnancy weight gain, food group classifications, other food ingredients and lifestyle issues, food safety issues, common problems associated with pregnancy, the benefits of fulfilling nutrient requirements during pregnancy and the consequences of maternal malnutrition, among other things. Third, providers discussed how to conduct nutrition education and counselling, as well as identify the important counselling skills, key messages and achievable actions and the ‘Greet, Ask, Listen, Identify, Discuss, Repeat, Appoint’ (GALIDRA) steps, which includes a checklist for how to counsel clients and reach an agreement. During this final session, all providers practised through case scenarios and role plays how to give pregnant women simple, personalized and specific recommendations on achievable actions for nutrition changes during pregnancy using the health belief model. All providers were then given supplementary materials such as training modules, job aids and summary pamphlets to use and display in their ANC units.

We evaluated the training by the results of pre-post test questions given to the participants based on the training module. We discussed areas that ANC providers seem to be weak in after the post-test in order to clarify any misunderstandings throughout the sessions. ANC providers implemented the training in their ANC units immediately after the training. We provided ANC providers of intervention health centres with everyday supportive supervision, which lasted for one week after the training to help identify and solve any difficulties while implementing the training and fill missing gaps if any.

### Data collection and variables

Data were collected from all ANC providers at pre-intervention (baseline) and post-intervention (follow-up). Follow-up data were collected from the same ANC providers 20 weeks after baseline. Three consecutive ANC consultations were observed from each ANC provider (*n* = 240 observations, 80 providers) at each stage of data collection. A total of 480 observations were collected both at baseline and follow-up. All outcome variables were assessed using an observation checklist adapted from BINLM and other literatures (Marías and Glasauer, 2014; Federal Ministry of Health Ethiopia, 2016a; Zelalem *et al.*, 2017). The survey tool was pre-tested among ANC providers from health centres not included in the study. Trained health professionals who were not working in the selected health centres conducted each stage of data collection. Data include the following.

#### Baseline characteristics of ANC providers

Baseline characteristics of ANC providers such as age, sex, field of study, educational status, institute of graduation educational status, years of experience, previous nutritional training and self-reported confidence in providing nutritional counselling to pregnant women (measured as ‘Confident’, ‘Moderately confident’ and ‘Not confident’). Baseline information was collected once during the baseline.

#### Outcome variables

Overall, outcomes are presented in two forms: as yes/no outcomes (for a: patient engagement and b: nutritional messaging) and scores (for c: more quantifiable nutritional counselling skills).

- *ANC providers’ engagement with the client* includes greeting the client, listening, showing interest, identifying key problems, avoiding judging words while speaking, praising the woman, recommending achievable actions, checking if the woman has understood the recommendations, measuring weight, monitoring gestational weight gain, taking client history and scheduling follow-up appointments. These variables were yes/no questions: ‘yes’ if the provider demonstrated the correct practice and ‘no’ if otherwise.
- Similarly, *nutritional messages delivered to pregnant women* include informing pregnant women about health and nutritional risks, the need for extra energy, one additional meal, minimum dietary diversity, daytime rest, gestational weight gain and early initiation of breastfeeding. These variables were also yes/no questions: ‘yes’ if the provider demonstrated the correct practice and ‘no’ if otherwise. For these yes/no outcomes, the above variables refer to the ANC having delivered the specific critical nutritional information to the woman during her visit. There is only one associated message, i.e. ANC providers should inform pregnant women about having one extra meal during pregnancy.
- In addition, other *more quantifiable nutritional messages* based on the recommended nutritional practice checklists such as IFA supplementation, food groups, iodine (iodized salt), consequences of undernutrition, things to avoid/limit during pregnancy and solutions to common problems during pregnancy were scored by fulfilling each item in the observation checklist per domain. Each of these nutritional domains had a set of guided checklists. For every item in the checklist per domain, a provider gets a score of 1 up to the maximum number of items assigned in that domain. Outcomes that are presented in scores contain more than one nutritional message in them, as described in Table 1, and are calculated per skill. Specifically, scores were calculated based on how many nutritional messages should have been included for each outcome of interest. Thus, a provider can achieve a score from zero up to the maximum allowable score for every nutritional counselling domain. For example, messages regarding IFA supplementation include four messages that pregnant women should have received from the provider during their visit. The ANC provider should inform pregnant women about taking IFA supplementation every day, its importance, taking the supplement after a meal and checking adherence to IFA supplementation. When observing providers during the ANC visit, we did not evaluate IFA supplement prescription because supplementations were offered during the start of ANC for the whole trimester, and not at every ANC visit. Table 1 details the definitions and associated maximum scores for each of the outcome variables.

**Table 1:** Definitions related to the quantifiable nutritional counselling skills of ANC providers, along with their maximum score points

Quantifiable nutritional messages (domains)	List of nutritional messages	Max. score points
Overall nutritional messages	Food groups, IFA supplementation, Consequences of under nutrition, Things to avoid/limit, Solutions to common problems during pregnancy, Iodine (iodized salt)	102
Food groups	Intake of recommended items for each of the following food groups: grains, pulses, meat and poultry, fish/sea food, dairy, eggs, fruit and vegetables, fats and oils	61
IFA supplementation	Discussed about taking IFA every day, its importance, taking the supplement after a meal, checking adherence to IFA supplementation	4
Consequences of under-nutrition	Discussed about maternal complications and foetal complications	11
Things to avoid/limit	Discussed avoidance of alcohol, excess caffeine and smoking	9
Solutions to common problems during pregnancy	Discussed how to address common problems during pregnancy such as nausea, vomiting, heartburn and constipation	11
Iodine (iodized salt)	Informed about use of iodized salt and discussed its importance, dietary sources of iodine, adding salt when serving food instead of while cooking the meal and proper storage of salt	6

### Statistical analysis

ANC consultations were the units of analysis in this study. The counselling approach of ANC providers was analysed from 480 observations of ANC consultations. We used descriptive statistics to summarize the baseline characteristics of ANC providers by allocation status.

We used mixed-effect linear regression to analyse the impact of the intervention on all outcomes. We included the interaction of allocation status (intervention and control) and time (baseline and follow-up) in the regression model. ANC providers and health centres were analysed as random effects in the regression model. A separate mixed-effect linear regression model was fitted for each outcome.

We presented difference-in-difference (DID) impact estimators (derived from the mixed-effect regression) for all outcomes to display the effect of the intervention. Since this is a randomized trial other approaches to analysis may be more powerful (Hooper *et al.*, 2018), but we prefer to use the DID impact estimator because of its familiarity and intuitive interpretation (Colombia University Mailman School of Public Health, 2011). For binary outcomes, the DID estimator is the difference between study arms in the change over time in the percentage reporting the outcome; for score outcomes the DID estimator is the difference in the change in the mean value of the outcome. DP (Follow-up result of an outcome—Baseline result of that outcome) are also presented along with the DID estimates. *P*-values and 95% confidence intervals of each outcome were also derived from the regression model.

All analyses were adjusted for institute of graduation (public vs private), field of study and educational status of ANC providers in the regression model. After the mixed-effect regression, we used Finner's adjusted test (Finner, 1993) to adjust for Type I error inflation that may result from the multiple testing from the single study/dataset. All *P*-values in each table (Tables 3–5) were entered in WinPepi software version 11.65 to derive Finner's adjusted *P*-values. Collected data were coded and entered using EPI data version 4.2.0. Data were analysed using Stata version 14.0.

**Table 3:** Client experiences and client approach of ANC providers during observed ANC visits at both baseline and follow-up by intervention status

Client experiences and client approach		Intervention^c^		Control^d^		DID impactestimator^e^	95% CI^f^	*P*-value
		Freq.	%	Freq.	%	%		
Greeted the client								
	Baseline^a^	107	89.2	110	91.7	**5.8**	−4.23–15.90	**0.256**
	Follow-up^b^	119	99.2	115	95.8			
Measured weight								
	Baseline^a^	1	0.83	4	3.33	**9.1**	0.47–17.85	**0.001**
	Follow-up^b^	11	9.17	3	2.50			
Monitored gestational weight gain								
	Baseline^a^	2	1.67	0	0.00	**38.3**	26.43–50.22	**0.001**
	Follow-up^2^	48	40.00	0	0.00			
Listened to what the client had to say								
	Baseline^a^	119	99.2	120	100.0	**0.8**	0.75–2.42	**0.304**
	Follow-up^b^	120	100.0	120	100.0			
Showed interest in what the client said								
	Baseline^a^	94	78.3	107	89.2	**21. 6**	18.10–41.52	**0.032**
	Follow-up^b^	120	100.0	107	89.2			
Let her talk before responding								
	Baseline^a^	113	94.2	116	96.7	**5.8**	−0.89–12.55	**0.089**
	Follow-up^b^	120	100.0	116	96.7			
Gave praise for what the pregnant woman was doing right								
	Baseline^a^	27	22.5	10	8.3	**30.0**	10.60–49.39	**0.002**
	Follow-up^b^	61	50.8	8	6.7			
Avoided judging words while speaking								
	Baseline^a^	67	55.8	71	59.2	**35.8**	15.58–56.09	**0.001**
	Follow-up^b^	117	97.5	78	65.0			
Identified key difficulties the pregnant woman was having								
	Baseline^a^	31	25.83	30	25.00	**30.8**	11.54–50.12	**0.002**
	Follow-up^b^	65	54.17	27	22.50			
Discussed possible options to practice recommendations								
	Baseline^a^	8	6.7	2	1.7	**32.5**	19.99–45.00	**0.001**
	Follow-up^b^	50	41.7	5	4.2			
Recommended achievable actions								
	Baseline^a^	71	59.2	59	49.2	**31.6**	11.79–51.53	**0.002**
	Follow-up^b^	96	80.0	46	38.3			
Helped her agree and repeat achievable actions								
	Baseline^a^	1	0.83	1	0.8	**20.0**	8.20–39.17	**0.041**
	Follow-up^b^	24	20.0	0	0.0			

a Baseline *N* = 240;

b Follow-up *N* = 240;

c Baseline intervention Freq. = 120;

d Follow-up intervention Freq. = 120;

e Baseline follow-up Freq. = 120;

f Follow-up control Freq. = 120;

g DID impact estimator using mixed-effect linear regression with health centres and ANC providers as random effects; adjusted for health-care provider’s institute of graduation, field of study and educational status;

h CI, confidence interval. All *P*-values in this table were adjusted using Finner's adjusted test after the mixed-effect regression.

**Table 4:** Nutrition messages delivered by ANC providers to pregnant women during observed ANC consultations at both baseline and follow-up by intervention status

Nutrition messages		Intervention^c^		Control^d4^		DID impactestimator^e^	95% CI^f^	*P*-value	ICC^g^
		Freq.	%	Freq.	%	%			
Increased risk during pregnancy									
	Baseline^a^	27	22.50	28	23.33	60.8	42.59–79.07	0.001	0.289
	Follow-up^b^	104	86.67	32	26.67				
The need for extra energy									
	Baseline^a^	44	36.67	47	39.17	58.3	42.54–74.12	0.001	0.142
	Follow-up^b^	118	98.33	51	42.50				
One additional meal									
	Baseline^a^	61	50.83	68	56.67	50.0	27.78–72.21	0.001	0.57
	Follow-up^b^	120	100.00	67	55.83				
Minimum dietary diversity									
	Baseline^a^	5	4.17	6	5.00	75.0	59.62–90.37	0.001	0.198
	Follow-up^b^	92	76.67	3	2.50				
Daytime rest									
	Baseline^a^	43	35.83	40	33.33	51. 6	30.16–73.16	0.001	0.093
	Follow-up^b^	120	100.00	55	45.83				
Gestational weight gain									
	Baseline^a^	35	29.17	39	32.50	76. 6	59.94–93.39	0.001	0.255
	Follow-up^b^	117	97.50	29	24.17				
Early initiation of breastfeeding									
	Baseline^a^	28	23.33	18	15.00	-2.5	-2.41–1.91	0.821	0.083
	Follow-up^b^	36	30.00	29	24.17				

a Baseline *N* = 240;

b Follow-up *N* = 240;

c Baseline intervention Freq. = 120; Follow-up intervention Freq. = 120;

d Baseline follow-up Freq. = 120; Follow-up control Freq. = 120;

e DID impact estimator using mixed-effect linear regression with health centres and ANC providers as random effects; adjusted for health-care provider’s institute of graduation, field of study and educational status;

f CI, Confidence interval;

g ICC, intra-cluster correlation coefficient. All *P*-values in this table were adjusted using Finner's adjusted test after the mixed-effect regression.

**Table 5:** Nutrition messages delivered by ANC providers to pregnant women during observed ANC consultations at both baseline and follow-up by intervention status

Nutrition messages		Intervention^3^		Control^4^		DID impact estimator^5^	95% CI^6^	*P*-value	ICC^8^
		Mean	SD	Mean	SD^7^	Mean score			
Overall nutritional messages									
*(Max 102 points)*	Baseline^1^	6.1	5.0	6.0	4.6	29.5	24.89–34.16	0.001	0.513
	Follow-up^2^	37.0	11.1	7.4	4.5				
IFA supplementation									
*(Max 4 points)*	Baseline^1^	1.4	0.8	1.5	0.8	1.7	1.46–2.03	0.001	0.423
	Follow-up^2^	3.3	0.7	1.6	0.7				
Food groups									
*(Max 61 points)*	Baseline^1^	2.5	2.9	2.2	2.4	15.9	13.33–18.51	0.001	0.511
	Follow-up^2^	19.1	6.6	2.8	2.4				
Consequences of under-nutrition									
*(Max 11 points)*	Baseline^1^	0.5	0.9	.35	0.8	4	3.27–4.87	0.001	0.368
	Follow-up^2^	4.7	2.3	0.4	0.8				
Things to limit/avoid									
*(Max 9 points)*	Baseline^1^	0.7	1.1	1.1	1.2	2.9	1.62–4.2	0.001	0.33
	Follow-up^2^	3.9	1.7	1.4	1.1				
Solutions to common problems during pregnancy									
*(Max 11 points)*	Baseline^1^	0.5	1.2	0.5	1.1	2.3	1.37–3.25	0.001	0.102
	Follow-up^2^	2.9	3.1	0.6	1.2				
Iodized salt									
*(Max 6 points)*	Baseline^1^	0.4	0.7	0.4	0.8	2.5	2.1–2.9	0.001	0.549
	Follow-up^2^	3.2	0.8	0.6	0.9				

1 Baseline *N* = 240;

2 Follow-up *N* = 240;

3 Baseline intervention Freq. = 120;

3 Follow-up intervention Freq. =120;

4 Baseline follow-up Freq. = 120;

4 Follow-up control Freq. = 120;

5 Average DID impact estimator using mixed-effect linear regression with health centres and ANC providers as random effects; adjusted for health-care provider’s institute of graduation, field of study and educational status;

6 CI, confidence interval;

7 SD, standard deviation;

8 ICC, intra-cluster correlation coefficient. All *P*-values in this table were adjusted using Finner's adjusted test after the mixed-effect regression.

## Results

### Participant characteristics

**Figure 2:**
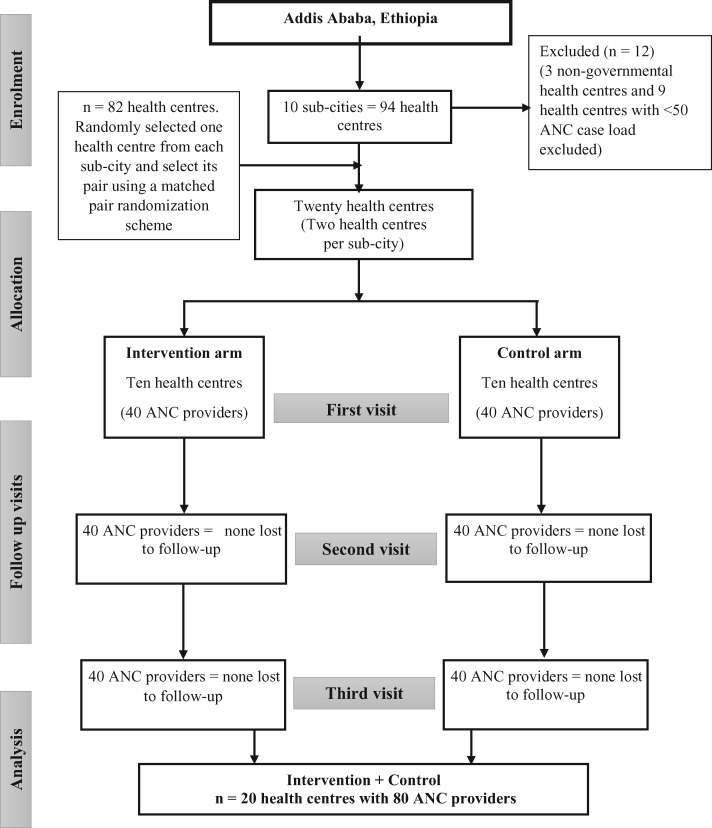
Flow diagram of the cluster health centres' and individual participant's enrolment, allocation and analysis. ANC, antenatal care

Twenty health centres and 80 ANC providers were included in the study with no losses to follow-up (Figure 2). Baseline characteristics of ANC providers were comparable for both intervention and control groups except for institute of graduation. The majority of ANC providers in the intervention arm had graduated from government schools (*n* = 35, 87.5%), while 25 (62.5%) ANC providers in the control arm graduated from private schools. Only 29 (36.25%) ANC providers stated that they felt confident in providing nutritional counselling to pregnant women, while 46 (57.5%) and five (6.25%) of them stated they were moderately confident and not confident, respectively (Table 2).

**Table 2:** Baseline sociodemographic characteristics of ANC providers by intervention status

Characteristics of ANC providers	**Intervention** ***n* ( 40 )**		**Control** ***n* ( 40 )**	
	*** n***	**%**	***n***	**%**
Age (in completed years)				
21–30	35	87.5	31	77.5
31–40	4	10.0	9	22.5
>40	1	2.5	0	0.0
Sex				
Male	7	17.5	9	22.5
Female	33	82.5	31	77.5
Marital status				
Single	28	70.0	25	62.5
Married/living together	11	27.5	14	35.0
Divorced/separated	0	0.0	1	2.5
Widowed	1	2.5	0	0.0
Field of study				
Midwife	29	72.5	24	60.0
Clinical nurse	4	10.0	3	7.5
BSc nurse	2	5.0	9	22.5
Health officer	5	12.5	4	10.0
Educational status				
Diploma	27	67.5	17	42.5
Degree	13	32.5	23	57.5
Institute of graduation				
Government	35	87.5	25	62.5
Private	5	12.50	15	37.5
Years of experience				
≤2	16	40.0	12	30.0
3–4	13	32.5	12	30.0
≥5	11	27.5	16	40.0
Previous nutritional training				
Yes	2	5.00	3	7.50
No	38	95.0	37	92.5
Self-reported confidence				
Not confident	3	7.5	2	5.0
Moderately confident	25	62.5	21	52.5
Confident	12	30.0	17	42.5
Think they will give better counselling with additional training				
Yes	35	87.5	33	82.5
No	5	12.5	7	17.5

Five (two from intervention and three from control) of the ANC providers stated that they had previous nutritional training specifically on management of acute malnutrition and nutritional assessment. Eighty-five per cent (43.75% from intervention and 41.25% from control) of ANC providers stated that they would be able to provide better nutritional counselling if additional training was given to them, while 15% stated otherwise (Table 2).

### Counselling skills of ANC providers

We found statistically significant improvement (*P* < 0.01) in the majority of the counselling skills of ANC providers (Tables 3–5).

#### ANC providers’ engagement with the client

ANC providers of the intervention arm were highly engaged with pregnant women during ANC consultations by identifying key difficulties the women were facing (DP intervention vs control) (DP 28.34% vs −2.5%; DID 30.8%), praising them for what they were doing right and avoiding judging words. There were also improvements in discussing available nutritional options to women (DP 35% vs 2.5%; DID 32.5%), recommending simple achievable actions on nutrition during pregnancy (DP 20.8% vs −10.9%; DID 31.6%) and making sure that the women understood by making them repeat doable actions. However, we did not find statistically significant changes on greeting the client, listening to her and letting her finish what she had to say before responding. All 80 ANC providers of both the intervention and control arms took the history of the client and scheduled the next appointment during both the baseline and follow-up study periods (Table 3).

#### Nutritional messages delivered to pregnant women

A similar improvement was also observed concerning ANC providers’ nutritional counselling skills, such as informing about one additional meal (DP 49.17% vs −0.84%; DID 50.0%) and about the risks of pregnancy, compared with the control arm. Significantly more ANC providers in the intervention arm informed pregnant women about daytime rest and minimum dietary diversity (DP 72.5% vs −2.5%; DID 75.0%). For those in the intervention arm, there was also significant improvement in counselling pregnant women on gestational weight gain (DP 68.33% vs −8.33%; DID 76.6%), monitoring gestational weight gain (DP 68.33% vs −8.33%; DID 76.6%) and measuring weight. However, there was no significant improvement in informing about early initiation of breastfeeding (DP 6.67% vs 9.17%; DID −2.5%) (Table 4).

#### Other more quantifiable nutritional messages delivered to pregnant women

On average, we found statistically significant improvement among ANC providers on the overall quantifiable nutritional counselling skills, as well as when informing pregnant women about IFA supplementation, food groups, consequences of under-nutrition, things to avoid/limit during pregnancy, solutions to common problems during pregnancy and iodized salt. None of the ANC providers in our study informed pregnant women about eating nuts either before or after the intervention (Table 5).

## Discussion

Overall, we found that the comprehensive in-service nutrition education and counselling training package is effective to improve ANC providers’ nutritional messaging to pregnant women. Our training package was designed by adapting the BINLM, and included training of ANC providers on basic pregnancy nutrition and counselling skills combined with distribution of modules, summary pamphlets, job aids and supportive supervision. Using such an approach, we found that ANC providers in the intervention arm gave more nutritional advice and had better communication skills than ANC providers in the control arm, highlighting the importance of designing comprehensive packages to improve the quality of ANC care.

Previous studies on in-service nutritional training interventions reported an increase in health-care providers’ counselling skills (Santos *et al.*, 2001; Pelto *et al.*, 2004; Zaman *et al.*, 2008) and improvement in the maternal/caregiver nutritional knowledge and dietary practice of pregnant women (Santos *et al.*, 2001; Pelto *et al.*, 2004; Zaman *et al.*, 2008; Sunguya *et al.*, 2013a,b; Nguyen *et al.*, 2017; Zelalem *et al.*, 2017).

Improving pregnancy nutrition via ANC is one of the critical components of health care interventions for maternal and child health endorsed by the government of Ethiopia (Federal Ministry of Health Ethiopia, 2005, 2010, 2013, 2015, 2016b). The current WHO ANC guideline also emphasizes the importance of nutrition interventions during pregnancy (World Health Organization, 2016).

In addition to ANC services, the methods and intervention design used in our study could be adapted to other interventions within the first 1000 days of life, such as on pregnancy nutrition, postnatal care, exclusive breastfeeding and complementary feeding. Studies documented the results of in-service training to be reflected in the observed improvement in maternal and caregiver nutritional knowledge, maternal dietary practices, exclusive breastfeeding, the birth weight of newborns, the infant feeding practice of caregivers, children’s daily energy intake, feeding frequency, dietary diversity and weight gain (Santos *et al.*, 2001; Pelto *et al.*, 2004; Zaman *et al.*, 2008; Sunguya *et al.*, 2013b; Nikièma *et al.*, 2017; Zelalem *et al.*, 2017). A systematic review also showed the effect of in-service nutrition training in improving the knowledge of health-care workers and their skills to manage child nutrition-related conditions such as under-nutrition (Sunguya *et al.*, 2013a). Thus, researchers and policy makers could draw on our experience to design and implement interventions targeting the critical window of opportunity for both maternal and child health and development.

Almost all ANC providers of the intervention and control arms in our study greeted the client and listened to them during observed ANC consultations at both baseline and follow-up visits. Despite the component of training concerning the importance of early initiation of breastfeeding, we found no significant difference on advice about this between the intervention and control arms. Similar effects were reported in studies that have observed how providers advise women on early initiation of breastfeeding (Pelto *et al.*, 2004; Zaman *et al.*, 2008).

Further in this study, ANC providers of the intervention arm were consistently using the job aid provided to them as part of the intervention package to provide counselling to pregnant women during ANC consultations. Studies also showed that training health workers to provide job aid-supported counselling improved their ANC counselling, maternal understanding (Jennings *et al.*, 2010; Oka *et al.*, 2019) and postnatal care and counselling (Jennings *et al.*, 2015).

Our findings could provide insights on the effectiveness of nutritional training packages for ANC providers in other cities with similar contexts to Addis Ababa. Our training also followed the standard module and training guide content (Guyon *et al.*, 2015; Federal Ministry of Health Ethiopia, 2016a) and it was designed in a way that can be applicable anywhere in a health centre setting. Our intervention occurred during the usual health services and there was no interference with the counselling apart from the training; neither did the authors gave financial incentives for ANC providers of either arms.

The findings of this study also highlight the importance and effectiveness of adapting national guidelines and training guides for the local health facility context. The scale-up of the programme in other places, especially rural areas, could also help improve ANC providers’ engagement and nutritional advice to pregnant women.

The short period between baseline and follow-up assessments (20 weeks) in our study made it difficult for us to understand the sustainability of the impacts of in-service nutritional training and to decide whether/when additional training is required. The observed counselling skills of ANC providers might decrease after long-term measurement such as six months or a year. A cluster randomized trial in Pelotas, Brazil (Santos *et al.*, 2001) measured doctors’ performance in terms of child nutritional assessment and communication skills both immediately after training and after 180 days (six months). The study observed a worsening in doctors’ performance in both outcomes, although a significant improvement was observed when compared with the control group. Such findings indicate that health-care providers might benefit from a refresher training a certain amount of time after their first training. It is our recommendation for future studies to assess the effect of such interventions after a longer period of time to help further understand the sustainability of interventions and possibly provide evidence of the time period in which training interventions in different contexts maintain a significant impact.

Furthermore, we were not able to control for Hawthorne's effect in which ANC providers might have performed differently because they were aware that they were being observed. This might affect the generalizability of the overall results of our study to the wider ANC provider community. Future studies could evaluate the feasibility, acceptability and challenges of the intervention package from ANC providers using qualitative and quantitative methods.

## Conclusion

A comprehensive in-service nutrition education and counselling package improved how ANC providers engaged with pregnant women and delivered nutrition messages during ANC consultations. We recommend provision of comprehensive in-service nutritional training for providers working in ANC units. Including supportive supervision, preparation of ANC nutritional guidelines and job aids is important to provide adequate nutrition education to pregnant women by trained ANC providers. Guidelines and job aids could include key messages and doable actions and could be delivered guided by a behavioural change theory model.

## References

[czaa101-B1] Colombia University Mailman School of Public Health. 2011 *Difference-in-Difference Estimation* [Online]. https://www.publichealth.columbia.edu/research/population-health-methods/difference-difference-estimation, accessed 19 May 2020.

[czaa101-B2] Federal Ministry of Health Ethiopia. 2005 *Health Sector Development Program 2005/6-2009/10*. Federal Ministry of Health Ethiopia.

[czaa101-B3] Federal Ministry of Health Ethiopia. 2010 *Health Sector Development Program 2010/11–2014/15*. Federal Ministry of Health Ethiopia.

[czaa101-B4] Federal Ministry of Health Ethiopia. 2013 *National Nutrition Programme 2013 (NNP I)*. Federal Ministry of Health Ethiopia.

[czaa101-B5] Federal Ministry of Health Ethiopia. 2015 *Health Sector Transformation Plan (HSTP) 2015-2020*. Federal Ministry of Health Ethiopia.

[czaa101-B6] Federal Ministry of Health Ethiopia. 2016a. *Blended and Integrated Nutrition Learning Module (BINLM), 2016*. Federal Ministry of Health Ethiopia.

[czaa101-B7] Federal Ministry of Health Ethiopia. 2016b. *National Nutrition Programme 2016 (NNP II)*. Federal Ministry of Health Ethiopia.

[czaa101-B8] Federal Ministry of Health Ethiopia. 2017 *Health and Health Related Indicators 2017/18*. Federal Ministry of Health Ethiopia.

[czaa101-B9] Finner H. 1993 On a monotonicity problem in step-down multiple test procedures. Journal of the American Statistical Association 88: 920–3.

[czaa101-B10] Guyon A, Quinn V, Nielsen J, Stone-Jimenez M 2015 Essential Nutrition Actions and Essential Hygiene Actions Training Guide: Health Workers and Nutrition Managers.*Washington, DC, The CORE Group*.

[czaa101-B11] Hooper R, Forbes A, Hemming K, Takeda A, Beresford LJB. 2018 Analysis of cluster randomised trials with an assessment of outcome at baseline. BMJ 360: k1121, 29559436 10.1136/bmj.k1121

[czaa101-B12] Jennings L, Yebadokpo A, Affo J, Agbogbe M. 2015 Use of job aids to improve facility-based postnatal counseling and care in rural Benin. Maternal and Child Health Journal 19: 557–65. 24916207 10.1007/s10995-014-1537-5

[czaa101-B13] Jennings L, Yebadokpo AS, Affo J, Agbogbe M. 2010 Antenatal counseling in maternal and newborn care: use of job aids to improve health worker performance and maternal understanding in Benin. BMC Pregnancy and Childbirth 10: 75. 21092183 10.1186/1471-2393-10-75 PMC3002891

[czaa101-B14] Marías Y, Glasauer P. 2014 Guidelines for Assessing Nutrition-Related Knowledge, Attitudes and Practices. Food and Agriculture Organization of the United Nations, Rome (FAO).

[czaa101-B15] Moore Q, Johnson A. 2015. Best practices for using health education to change behavior. James A. Baker III Institute for Public Policy of Rice University.

[czaa101-B16] Nguyen PH, Kim SS, Sanghvi T et al 2017 Integrating nutrition interventions into an existing maternal, neonatal, and child health program increased maternal dietary diversity, micronutrient intake, and exclusive breastfeeding practices in Bangladesh: results of a cluster-randomized program evaluation. The Journal of Nutrition 147: 2326–37. 29021370 10.3945/jn.117.257303 PMC5697969

[czaa101-B17] Nikièma L, Huybregts L, Martin-Prevel Y et al 2017 Effectiveness of facility-based personalized maternal nutrition counseling in improving child growth and morbidity up to 18 months: a cluster-randomized controlled trial in rural Burkina Faso. PLoS One 12: e0177839. 28542391 10.1371/journal.pone.0177839 PMC5444625

[czaa101-B18] Oka M, Horiuchi S, Shimpuku Y, Madeni F, Leshabari S. 2019 Effects of a job aid-supported intervention during antenatal care visit in rural Tanzania. International Journal of Africa Nursing Sciences 10: 31–7.

[czaa101-B19] Pelto GH, Santos I, GonçAlves H et al 2004 Nutrition counseling training changes physician behavior and improves caregiver knowledge acquisition. The Journal of Nutrition 134: 357–62. 14747672 10.1093/jn/134.2.357

[czaa101-B20] Santos I, Victora CG, Martines J et al 2001 Nutrition counseling increases weight gain among Brazilian children. The Journal of Nutrition 131: 2866–73. 11694610 10.1093/jn/131.11.2866

[czaa101-B21] Sunguya B, Poudel KC, Mlunde LB, Urassa DP. 2013a. Nutrition training improves health workers’ nutrition knowledge and competence to manage child undernutrition: a systematic review.*Frontiers in Public Health*, *1*, p.37. 10.3389/fpubh.2013.00037 PMC3859930 24350206

[czaa101-B22] Sunguya BF, Poudel KC, Mlunde LB et al 2013b. Effectiveness of nutrition training of health workers toward improving caregivers’ feeding practices for children aged six months to two years: a systematic review. Nutrition Journal 12: 66. 23688174 10.1186/1475-2891-12-66 PMC3668136

[czaa101-B23] Taylor D, Bury M, Campling N et al 2006 A Review of the Use of the Health Belief Model (HBM), the Theory of Reasoned Action (TRA), the Theory of Planned Behaviour (TPB) and the Trans-Theoretical Model (TTM) to Study and Predict Health Related Behaviour Change. London, UK: National Institute for Health and Clinical Excellence, 1–215.

[czaa101-B24] The DHS Program ICF Ethiopia. 2016. Ethiopian Demographic and health survey 2016: key indicators report.

[czaa101-B25] Veena SR, Gale CR, Krishnaveni GV et al 2016 Association between maternal nutritional status in pregnancy and offspring cognitive function during childhood and adolescence; a systematic review. BMC Pregnancy and Childbirth 16: 220. 27520466 10.1186/s12884-016-1011-z PMC4982007

[czaa101-B26] Wilkinson SA, Tolcher D. 2010 Nutrition and maternal health: what women want and can we provide it? Nutrition & Dietetics 67: 18–25.

[czaa101-B27] Williamson C. 2006 Nutrition in pregnancy. Nutrition Bulletin 31: 28–59.

[czaa101-B28] World Health Organization (WHO). 2016 WHO recommendations on antenatal care for a positive pregnancy experience.World Health Organization, Geneva. https://apps.who.int/iris/bitstream/handle/10665/250796/9789241549912-eng.pdf, accessed 3 November 2020. 28079998

[czaa101-B29] Wu G, Bazer FW, Cudd TA, Meininger CJ, Spencer TE. 2004 Maternal nutrition and fetal development. The Journal of Nutrition 134: 2169–72. 15333699 10.1093/jn/134.9.2169

[czaa101-B30] Zaman S, Ashraf RN, Martines J. 2008 Training in complementary feeding counselling of healthcare workers and its influence on maternal behaviours and child growth: a cluster-randomized controlled trial in Lahore, Pakistan. Journal of Health, Population, and Nutrition 26: 210–22. PMC2740673 18686554

[czaa101-B31] Zelalem A, Endeshaw M, Ayenew M, Shiferaw S, Yirgu R. 2017 Effect of nutrition education on pregnancy specific nutrition knowledge and healthy dietary practice among pregnant women in Addis Ababa. Clinics in Mother and Child Health 14: 265.

